# 

**Published:** 1990-12

**Authors:** 


South West Vascular Surgeons

### WEST OF ENGLAND MEDICAL JOURNAL

**DECEMBER 1990** ISSN: 0960 6440
**Volume 105(iv)**

Copyright © West of England Medical Journal Co Ltd

**Contents**

102 **On the Teaching of Operative Surgery**
Ronald Belsey
103 **Teaching and Research of the Art of Medicine**
Andrew J. Cave
105 **Book Reviews**
Travels of a Surgeon. Surgical Journeys
106 **Bayes Theorem for the Clinician**
G. H. Hall and A. P. Round
110 **Fellowship of The Royal College of General Practitioners by Assessment**
Denis Pereira Gray
111 **Imaging of Intracranial Tumours—Comparison of Computed Tomography and Magnetic Resonance Imaging**
M. Kvriakose
114 **Plancental Abruption following Vaginal Administration of Prostoglandin E2 for Induction of Labour**
R. Kulkarni, K. Hawkins and A. H. W. Boyle
116 **An Arts Co-ordinator in a Psychiatric Hospital**
D. S. Allen and S. Hosking
117 **Mummification in Ancient Egypt**
Joseph Sluglett
120 **Soap Gets in Your Eyes**
Alistair Laidlaw and Philip Bloom
121 **From Our Correspondents—To ski or not to ski on replaced hips**
Prognosis by Prophecy—A Memoir of Carl Jung
123 **First Bristol Radiology Course, November 1989**
124 **South West Obstreticians and Gynaecologists Society, May 1990 Meeting**
126 **Surgical Club of the South West, May and October 1990 Meetings**
128 **South West Orthopaedic Club, Spring 1990 Meeting**
130 **South West Vascular Surgeons, February 1990 Meeting**
132 **Index Volume 105**

***What I know remains unknown, unless by me to others shown.***
**Lucilius (180–102 BC).**

